# Post-Synthetic Surface Quaternization of Agarose Cryogels: Optimizing Structural Integrity and Broad-Spectrum Growth Inhibition

**DOI:** 10.3390/gels12090792

**Published:** 2026-09-01

**Authors:** Ahmet Erdem, Elif Beyza Eren, Farouk Segujja, Elif Kale Bakir, Yonca Yuzugullu Karakus, Suheda Ercek, Tugba Dispinar Gezer

**Affiliations:** 1 Department of Biomedical Engineering, Faculty of Technology, Kocaeli University, 41001 Kocaeli, Turkey; 2 TUBITAK UME, TUBITAK, Gebze Yerleşkesi, Gebze, 41470 Kocaeli, Turkey; 3 Department of Biology, Faculty of Science and Arts, Kocaeli University, 41001 Kocaeli, Turkey

**Keywords:** agarose cryogel, surface cationization, quaternary ammonium, antibacterial activity, chronic wound care

## Abstract

Agarose is a promising biopolymer for wound healing due to its biocompatibility and ability to form stable macroporous cryogels. However, its bioinert nature limits its antibacterial functionality, while conventional chemical modification can disrupt its macroporous architecture and mechanical integrity. Here, we present a versatile post-synthetic heterogeneous surface quaternization strategy to transform prefabricated agarose cryogels into surface-modified agarose cryogels (SMACs) with contact-active antibacterial functionality while preserving their interconnected supermacroporous architecture. Using 3-chloro-2-hydroxypropyltrimethylammonium chloride (CHPTAC) in an alkaline environment, quaternary ammonium moieties were covalently immobilized onto the agarose surface. A multivariable optimization approach examining reaction time, temperature, and scaffold concentration yielded surface quaternization values (DS_EA_) of 0.05 and 0.11 for SMAC-4 and SMAC-24, respectively, with successful modification confirmed via SEM-EDX and FT-IR analyses. Quaternization altered the physicochemical and mechanical properties of the scaffolds; notably, the average porosity increased from 66.7% in AC-0 to 78.7% in SMAC-24 while maintaining the integrity of the porous structure. Biological evaluation against *Escherichia coli* and *Staphylococcus aureus* identified SMAC-24 as the most favorable formulation, exhibiting the strongest antibacterial activity and maintaining 88.1 ± 1.6% human umbilical vein endothelial cell (HUVEC) viability after 24 h of exposure to scaffold extracts. These findings demonstrate that post-synthetic surface quaternization provides agarose cryogels with contact-active antibacterial functionality and favorable cytocompatibility, highlighting their potential as non-leaching antibacterial agarose scaffolds for chronic wound care.

## 1. Introduction

Chronic wounds, burn injuries, and non-healing surgical incisions represent an escalating global healthcare burden, affecting millions of patients and incurring multi-billion-dollar clinical expenditures annually [1]. The physiological cascade of wound healing is a highly synchronized process involving hemostasis, inflammation, proliferation, and tissue remodeling [2]. However, this delicate sequence is frequently disrupted by localized bacterial colonization, leading to the formation of resilient biofilms that trigger persistent inflammatory responses and severely delay dermal regeneration. To mitigate these complications, wound care materials have transitioned from passive, traditional coverages, such as cotton gauzes and nonwoven bandages, which suffer from poor fluid management and cause secondary mechanical trauma upon removal, toward highly absorptive, biocompatible polymeric materials [3]. An ideal wound dressing must exhibit a multifaceted performance profile, including maintaining a localized moist microenvironment, facilitating unhindered gaseous exchange, possessing excellent elastomeric compliance, demonstrating a high capacity for wound exudate management, and crucially, exerting broad-spectrum antimicrobial defense without inducing cytotoxic leakage into the regenerating tissue bed [4].

To achieve these rigid structural and functional prerequisites, three-dimensional polymeric scaffolds, namely hydrogels, have been at the forefront of biomaterials engineering [5]. Among these architectures, cryogels fabricated via cryotropic gelation at subzero temperatures present distinct physical advantages over conventional crosslinked hydrogels. Conventional hydrogels typically exhibit dense, nanoporous mesh structures that inherently restrict water transport kinetics and limit the accommodation of highly viscous cellular exudates. In contrast, cryogels possess an extensively interconnected supermacroporous network, where the pore walls are established by the cryoconcentration of polymeric chains around microscale ice crystal templates. Upon thawing, these sacrificial ice templates vacate a continuous path of macropores ranging from tens to hundreds of micrometers. This distinct morphology yields sponge-like reversible compressibility, shape memory retention, and exceptionally rapid swelling kinetics [6,7]. Among the natural polymers used as cryogel backbones, agarose, a marine-derived linear polysaccharide composed of alternating units of agarobiose, a disaccharide composed of d-galactose and 3,6-anhydro-l-galactopyranose connected by alpha-1,3 and beta-1,4 glycosidic linkages, holds immense clinical prominence owing to its excellent biocompatibility, low immunogenicity, non-toxicity, and thermoreversible physical gelation governed by extensive interchain hydrogen bonding [8]. Nevertheless, native agarose is inherently non-ionic and completely lacks intrinsic functional groups capable of preventing bacterial adhesion or neutralizing pathogens, rendering pure agarose cryogels highly vulnerable to opportunistic microbial proliferation at clinically compromised wound sites.

To impart robust antimicrobial properties without relying on the encapsulation of antibiotics or heavy metal nanoparticles, which are prone to uncontrolled burst release, rapid depletion, and systemic cytotoxicity [9], the covalent grafting of permanent positive charges onto the polysaccharide backbone represents a highly sustainable model [10,11]. Cationization through the introduction of quaternary ammonium groups, such as those derived from 3-chloro-2-hydroxypropyltrimethylammonium chloride (CHPTAC), transforms the inert polysaccharide into an electrostatically active derivative. Localized quaternary ammonium moieties provide a permanent positive surface charge density that remains stable regardless of the surrounding physiological conditions [12]. When pathogenic bacterial cells come into direct contact with the modified matrix, the cationic sites establish irreversible electrostatic interactions with the negatively charged molecules of the bacterial envelope, specifically, lipopolysaccharides in Gram-negative strains and teichoic acids in Gram-positive strains. This electrostatic binding creates a localized osmotic displacement, leading to the physical disruption of the lipid bilayer, leakage of intracellular components, and subsequent cell lysis through a non-leaching, contact-killing mechanism [13].

The conventional approach for the synthesis of such cationic polysaccharides involves homogeneous-phase reactions, where agarose is modified in its fully dissolved sol state [14,15]. However, this pre-modification methodology may induce significant technical drawbacks that compromise the structural integrity of the final product. The substitution of bulky, hydrophilic quaternary ammonium groups onto the agarose chain may alter the natural viscoelastic behaviors of macromolecules, negatively impacting the natural helix formation mechanism of agarose, changing its coil-to-helix transition temperature, and interfering with the nucleation growth of ice crystals during sub-zero freezing [16]. Moreover, the development of a controlled post-functionalization strategy allows for the systematic targeting of a highly selective biophysical equilibrium. By carefully regulating the surface modification, it is possible to achieve a distinct charge density that provides a potent electrostatic force to disrupt bacterial envelopes while remaining exceptionally far below the cytotoxicity threshold for human fibroblast and keratinocyte membranes [17]. A multivariable experimental design can be strategically established to elucidate the coupled kinetic effects of reaction parameters on the final surface charge parameters of polysaccharide matrices. In designing such platforms, balancing the positive charge density with cellular compatibility is a vital objective for clinical safety. Indeed, it has been shown that the extent and type of cationic modification can significantly affect the physicochemical and biological properties of polysaccharide-based biomaterials [15,18]. However, cytotoxic responses depend on the polymer chemistry, charge density, and experimental conditions; thus, cytocompatibility should be experimentally confirmed for each material system [18].

To overcome the potential destructive morphological alterations during the cryogelation process of cationized agarose precursors, this study considers a post-functionalization strategy designed specifically for pre-formed, dried agarose cryogel scaffolds. The distinctive architectural morphology of cryogels plays a foundational role in dictating the feasibility and efficiency of this heterogeneous approach [19,20]. While traditional hydrogels impose severe steric hindrances that confine chemical reactions strictly to the outer boundaries, the highly interconnected supermacropores of cryogels function as open microchannels. This open-cell topology allows for unhindered capillary-driven infiltration of the liquid reaction phase (containing CHPTAC and NaOH) deep into the core of the prefabricated solid matrix, converting the voluminous macropores into localized reaction microchambers. This structural accessibility ensures that the etherification occurs uniformly throughout the internal pore walls without altering the macrostructural framework. The chemical structures, macroporous morphologies, mechanical stabilities, swelling behaviors, and degradation profiles of the cationized agarose cryogels were comprehensively characterized to map out the underlying structure–property relationships. Concurrently, their contact-killing antibacterial efficacy against model Gram-positive (*Staphylococcus aureus*) and Gram-negative (*Escherichia coli*) pathogens was systematically evaluated. By correlating the reaction time with the degree of surface functionalization, this study established the reaction time as a practical design parameter for regulating the balance between antibacterial efficacy and scaffold functionality in physically crosslinked agarose cryogels.

## 2. Results and Discussion

### 2.1. Synthesis Strategy and Structural Rationale of Cationic Cryogels

Agarose, a marine-derived natural polysaccharide harvested from red algae, is chemically constituted by the sequential alternative repetition of beta-d-galactopyranose and 3,6-anhydro-alpha-l-galactopyranose units [21]. This unique macromolecular architecture enables the polymer chains to completely dissolve in hot aqueous media by disrupting the extensive intra- and interchain hydrogen bonding interactions and subsequently assemble into thermally reversible, physically crosslinked hydrogels through network formation driven by double-helix bundling upon cooling. Taking advantage of this feature, physical cryotropic gelation was successfully employed in this study using an ice-templating mechanism. During the sub-zero freezing regime, the progressive nucleation and growth of ice crystals act as natural sacrificial templates, forcing the localized agarose chains to undergo cryo-concentration within the remaining micro-phases of the unfrozen bound water [22]. Following the removal of these ice templates upon melting after cryogelation, a continuous, mechanically stable, and highly interconnected supermacroporous network (designated as native agarose cryogel, AC-0) was generated. The abundance of primary and secondary hydroxyl (–OH) functional groups protruding from the agarose backbone renders these porous matrices highly susceptible to chemical modifications [15].

When designing such functionalized platforms, executing the cationization reaction in the fluid sol state prior to the cryotropic gelation process may present distinct theoretical challenges. Polysaccharide functionalization with bulky, hydrophilic quaternary ammonium groups at the macromolecular level is expected to disrupt the subsequent coil-to-helix transition dynamics of agarose chains upon cooling. Because physical agarose gelation is governed by strict spatial alignments and hydrogen-bonding networks, such premature modifications could theoretically impair the nucleation growth of ice crystal templates, posing a risk of yielding structurally compromised or poorly interconnected cryogels. To overcome these potential structural limitations, a post-synthetic heterogeneous surface functionalization strategy was implemented on the prefabricated, dry AC-0 scaffolds. The preformed cryogels were subjected to surface etherification using 3-chloro-2-hydroxypropyltrimethylammonium chloride (CHPTAC) in an alkaline environment to yield surface-quaternized derivatives (SMAC series in Table 1 and Table S1). This post-synthetic strategy ensures that the voluminous interconnected macroporous architecture remains intact while permanently localizing the active quaternary ammonium groups exclusively along the solid pore walls.

To systematically investigate the coupled effects of mass transport kinetics and thermodynamic stability on the physically crosslinked network, heterogeneous modifications were conducted across a multivariable matrix. Accordingly, the reactions were carried out for varying durations: at room temperature for 4–24 h (Table 1) and at 50 °C for 1–6 h (Table S1). In physically crosslinked systems, maintaining the etherification at room temperature (as performed for SMAC-4, SMAC-6, and SMAC-24) serves as a conservative design pathway to ensure ultimate morphological preservation against chemical dissolution [6,22,23,24]. Concurrently, elevated thermal inputs up to 50 °C (as explored for SMAC-1 to SMAC-6) were systematically evaluated to map the boundary thresholds where accelerated reaction kinetics and enhanced degrees of substitution (DS_EA_) intersect with the mechanical continuity and integrity of porous agarose networks. While elevated thermal regimes accelerate etherification kinetics and drive higher nitrogen accumulation, a crucial structure–property trade-off was identified regarding the mechanical sustainability of cryogels. The cryogel formulations post-functionalized at 50 °C (SMAC-1a50 to SMAC-6c50) exhibited a macroscopic loss in mechanical robustness, manifesting lower compressive resistance and enhanced brittleness than the native cryogel (AC-0). This thermal and chemical weakening of the physically crosslinked framework can be interpreted using three phenomenological mechanisms [25,26]. First, because the structural integrity of agarose cryogels is entirely sustained by thermoreversible non-covalent hydrogen bonds, maintaining the heterogeneous matrix at 50 °C inside an aqueous medium approaches the pre-sol transition zone, inevitably inducing partial thermal relaxation and microscopic loosening of the double-helix bundles within the pore walls. Second, this thermal destabilization is highly synergistic with the high concentration of the alkaline catalyst (NaOH). The synergetic effect of heat and alkali promotes localized macromolecular erosion, driving the minor leaching of uncrosslinked polysaccharide fragments into the reaction fluid and systematically thinning the load-bearing pore skeletons. Third, as quantitatively validated by the elemental matrix, a high thermal input triggers a rapid influx of cationic quaternary ammonium segments. The dense confinement of permanent, positive charges localized along the pore walls generates severe intra-strut electrostatic repulsions. These localized repulsive fields force adjacent agarose segments to push apart, disrupting the dense hydrogen-bonding network and significantly reducing the elasticity of the solid framework. Consequently, these mechanical limitations at 50 °C strongly validate the strategic necessity of the room-temperature optimization pathway (SMAC-4 to SMAC-24). By dampening the thermal energy, the room-temperature heterogeneous approach successfully circumvents both thermal relaxation and alkaline erosion, establishing a well-preserved macromolecular assembly where an optimal biological charge density (N (%) = 0.90) perfectly coexists with the highly resilient, sponge-like mechanical compliance required for advanced chronic wound care configurations. Based on these crucial physical and structural findings, SMAC-4 and SMAC-24 were exclusively selected as the representative quaternized formulations for all subsequent characterization, morphological assessment and biological evaluations. Although SMAC-6 exhibited well-preserved macroscopic integrity under room-temperature conditions, its nitrogen content demonstrated negligible variance compared to that of SMAC-4. To avoid data redundancy and maintain a clear, contrast-rich comparative framework, SMAC-6 was excluded from testing. Consequently, the remainder of this study focuses on the performance profiles of the low-charge resilient matrix (SMAC-4) and the high-charge optimized matrix (SMAC-24) against the native agarose scaffold (AC-0).

### 2.2. Elemental Analysis and Optimization of Heterogeneous Quaternization

The successful implementation of the post-synthetic heterogeneous etherification strategy on prefabricated agarose cryogels was quantitatively verified and tracked via combustion elemental analysis. As summarized in Table 1, native unmodified agarose exhibited a baseline nitrogen content of 0.00%, serving as an ideal nitrogen-free control matrix. Upon alkaline activation and subsequent reaction with CHPTAC, a significant and controllable increase in both the nitrogen weight percentage and the corresponding degree of substitution was observed across all modified SMAC samples, confirming the permanent immobilization of quaternary ammonium moieties onto the porous matrix. A comparative kinetic evaluation revealed that the reaction temperature was the primary driving force governing the rate of heterogeneous etherification. Under identical reactant concentrations and feeding ratios, at room temperature, the modification progressed at a slow rate, yielding an N ratio of only 0.41% after 4 h (SMAC-4) and reaching 0.49% at 6 h (SMAC-6). Even when the processing time was drastically extended to 24 h (SMAC-24), the nitrogen density was capped at 0.90% (DS_EA_ = 0.11).

Conversely, increasing the temperature to 50 °C accelerated the reaction kinetics. At 50 °C, a reaction time of just 2 h achieved a %N of 0.94% (SMAC-2c50), outperforming the 24 h room temperature run. Extending the thermal processing to 4 h and 6 h at 50 °C increased the nitrogen levels to 1.09% (SMAC-4c50) and a maximum value of 1.18% (SMAC-6c50), respectively. This thermodynamic contrast can be rationalized by the dual role of heat in heterogeneous polymer modifications. Elevated temperatures enhance the diffusion coefficients of the mobile reactants (NaOH and CHPTAC) within the liquid phase, facilitating their rapid convective and capillary transport into the solid agarose pore walls. Concurrently, 50 °C provides the necessary thermal activation energy to overcome the steric barriers of the tightly packed crystalline helical aggregates of the crosslinked agarose wall structure, rendering the buried primary hydroxyl (–OH) groups temporarily accessible for deprotonation and nucleophilic substitution. Furthermore, higher temperatures accelerate the in situ cyclization of CHPTAC into the highly reactive epoxide intermediate (EPTAC), thereby accelerating the overall etherification rate before competing hydrolysis reactions deplete the precursor.

The effect of reaction duration was systematically monitored at 50 °C (Table S1). As the reaction time increased from 1 h to 2 h and finally to 6 h, a steady, almost linear increase in nitrogen density (%N) was recorded at 0.70%, 0.94%, and 1.18% for SMAC-1c50, SMAC-2c50, and SMAC-6c50, respectively. This continuous progression demonstrates that the open, interconnected convective supermacropores of the cryogel network effectively act as continuous microchambers, preventing local reactant starvation and maintaining a steady concentration gradient across the solid–liquid interface over time. However, the kinetic rate began to exhibit a deceleration profile at the 6 h mark. Interestingly, variations in the initial cryogel scaffolding mass density (1, 2, and 4 mg/mL) consistently affected the functionalization efficiency. At a fixed temperature (50 °C) and duration (1 h), increasing the cryogel concentration of SMAC-1 formulations from 1 to 4 mg/mL resulted in a minor increase in nitrogen accumulation (0.64% to 0.70%). The same positive trend was observed at the 2 h interval for SMAC-2a50 to SMAC-2c50, where the %N increased from 0.80% to 0.94%. This phenomenon indicates that a higher scaffold concentration per unit volume provides a more compact microscale alignment of the pore walls, reducing the dead-volume channels and maximizing the effective collision frequency between the alkaline-activated hydroxyl sites and flowing cationic intermediates.

The reaction temperature seems to act as a primary driving force governing the rate of heterogeneous etherification. A multi-variable optimization matrix was established to investigate the effects of post-functionalization time and CHPTAC feeding conditions on the nitrogen content (%N) and degree of substitution determined via elemental analysis (DS_EA_) of the agarose cryogels. Unlike conventional homogeneous agarose cationization protocols that require elevated temperatures to prevent premature physical gelation [15], this study shows that the cationization of the pre-formed agarose cryogel matrices can successfully be carried out under ambient conditions. This mild reaction environment preserved the structural integrity of the macroporous architecture while avoiding thermal polymer degradation.

Previous studies on cationic polymers have demonstrated that biological responses to positively charged macromolecules depend on parameters including polymer concentration, exposure time, molecular weight, and cationic charge density [18]. In particular, cationic charge density and molecular weight were identified as important parameters governing interactions of polycations with cell membranes and subsequent cellular damage [18]. The initial interaction between cationic macromolecules and negatively charged cell membranes is mediated primarily by electrostatic interactions, while subsequent interactions with membrane components can disturb membrane structure and function. The degree of substitution obtained for SMAC-24 (DS_EA_ = 0.11) represents a controlled level of cationic functionalization rather than an intrinsically defined cytocompatibility threshold. This level of functionalization may provide a suitable basis for investigating the balance between the introduction of cationic groups and the biological compatibility of the polysaccharide scaffold. Nevertheless, the antibacterial efficacy and cytocompatibility of the SMAC cryogels have been studied experimentally to define DS_EA_ = 0.11 as an optimal biophysical equilibrium.

### 2.3. FTIR Spectroscopy and Chemical Modification Mechanism

**Figure 1:**
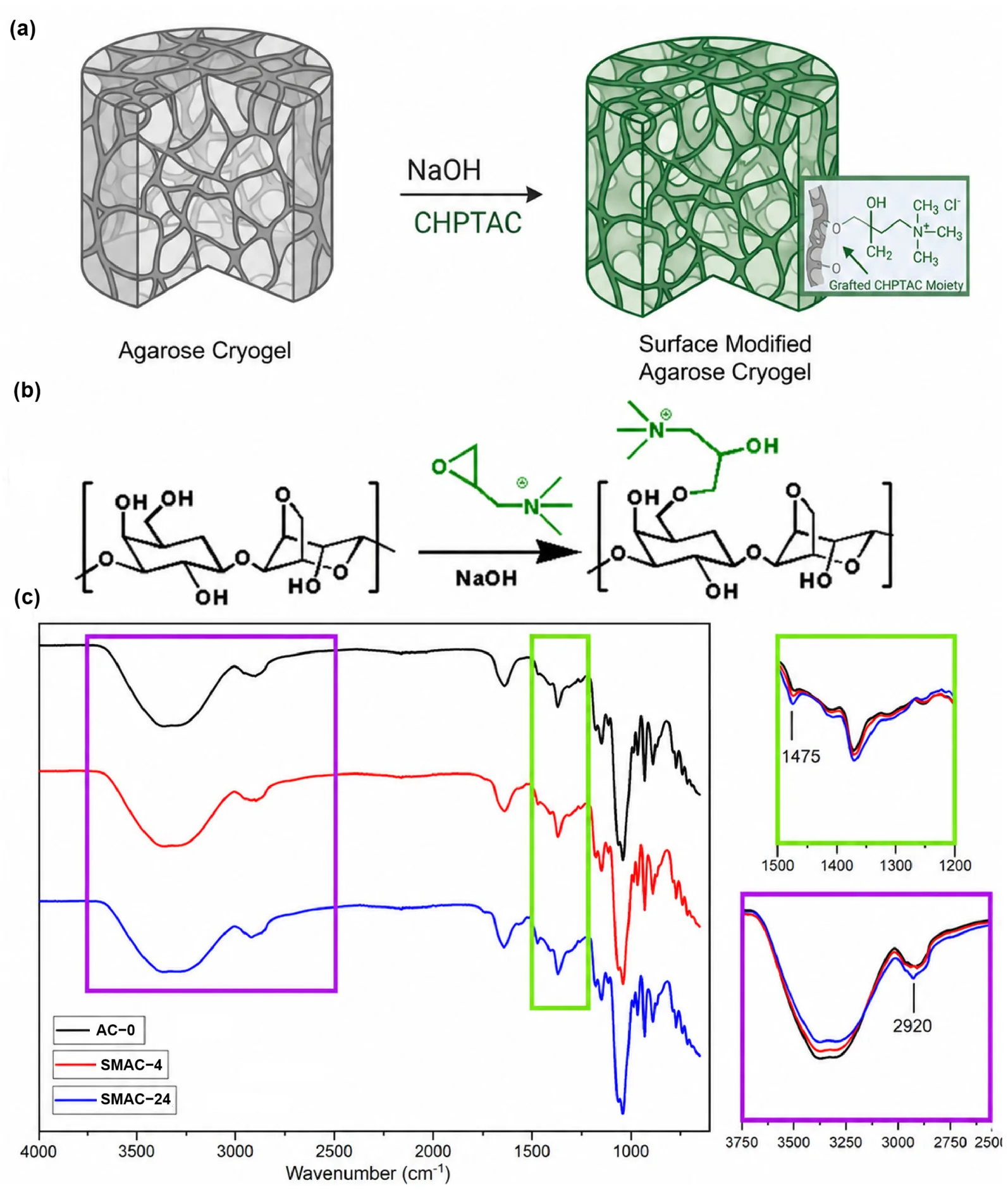
(**a**,**b**) Schematic illustration of the cationization of agarose cryogels with CHPTAC. (**c**) FTIR spectra of agarose cryogel (AC-0) and cationized agarose cryogels (SMAC-4 and SMAC-24).

The chemical structural alterations of the agarose cryogels were qualitatively validated using FTIR-ATR spectroscopy. Figure 1 chronologically illustrates a schematic representation of the heterogeneous surface modification (Figure 1a), the corresponding chemical reaction mechanism in an alkaline environment (Figure 1b), and the comparative FTIR spectra of the native AC-0, SMAC-4, and SMAC-24 scaffolds (Figure 1c). As shown in the reaction pathway (Figure 1b), the introduced NaOH catalyst promoted the in situ cyclization of the CHPTAC precursor into its highly reactive epoxide form, (2,3-epoxypropyl)trimethylammonium chloride (EPTAC). Concurrently, deprotonation of the primary hydroxyl groups localized on the galactopyranose units of the agarose backbone yields nucleophilic alkoxide ions. These active surface sites systematically attack the strained oxirane ring of the EPTAC intermediate via a nucleophilic ring-opening etherification mechanism, permanently anchoring the quaternary ammonium moieties exclusively along the solid pore walls (Figure 1a) [27].

The successful execution of this post-synthetic surface engineering framework was confirmed by tracking the characteristic vibrational fingerprints in the FTIR spectra (Figure 1c) [28,29]. All samples displayed baseline polysaccharide peaks inherent to the agarose backbone. The broad and intense absorption band spanning between 3600 cm^−1^ and 3200 cm^−1^ corresponds directly to the stretching vibrations of the intra- and intermolecularly hydrogen-bonded hydroxyl (–OH) groups. The distinct band appearing at approximately 2920 cm^−1^ is ascribed to the asymmetric stretching vibrations of the aliphatic C–H bonds in the carbohydrate framework. The fingerprint region below 1100 cm^−1^ resolves the characteristic glycosidic linkages and specific C–O stretching bands indicative of 3,6-anhydro-alpha-L-galactopyranose bridges.

Upon surface quaternization, distinct and systematic spectroscopic deviations emerged in the SMAC-4 and SMAC-24 profiles compared with those of the native AC-0 control. The most definitive evidence of successful covalent grafting is the emergence of a new sharp bending vibration band at 1475 cm^−1^. A subtle spectroscopic deviation at approximately 1475 cm^−1^, assigned to the asymmetric methyl bending of the quaternary ammonium substituent, was qualitatively detectable in the SMAC-4 and SMAC-24 profiles, consistent with the permanent immobilization of CHPTAC-derived cationic moieties on the agarose cryogel. However, the FTIR data are presented only as a qualitative line of evidence to corroborate the elemental and SEM-EDX analysis findings because of the inherently semi-quantitative nature of the FTIR-ATR intensities. Concurrently, a noticeable modification profile was observed in the C–H stretching region at 2920 cm^−1^. Following etherification, the intensity of this band exhibited relative suppression and broadening in the SMAC series. This phenomenon is attributed to the overlapping contributions of the newly introduced aliphatic spacer arms (-CH_2_CH(OH)CH_2_-) and the high density of hydrophilic quaternary head groups, which locally disturb the original structural packing and hydration state of the polysaccharide backbone. Taken together, these definitive FTIR shifts confirm that the room-temperature heterogeneous optimization framework effectively functionalizes the internal pore skeletons with high chemical control.

### 2.4. Swelling, Degradation, and Mechanical Properties

**Figure 2:**
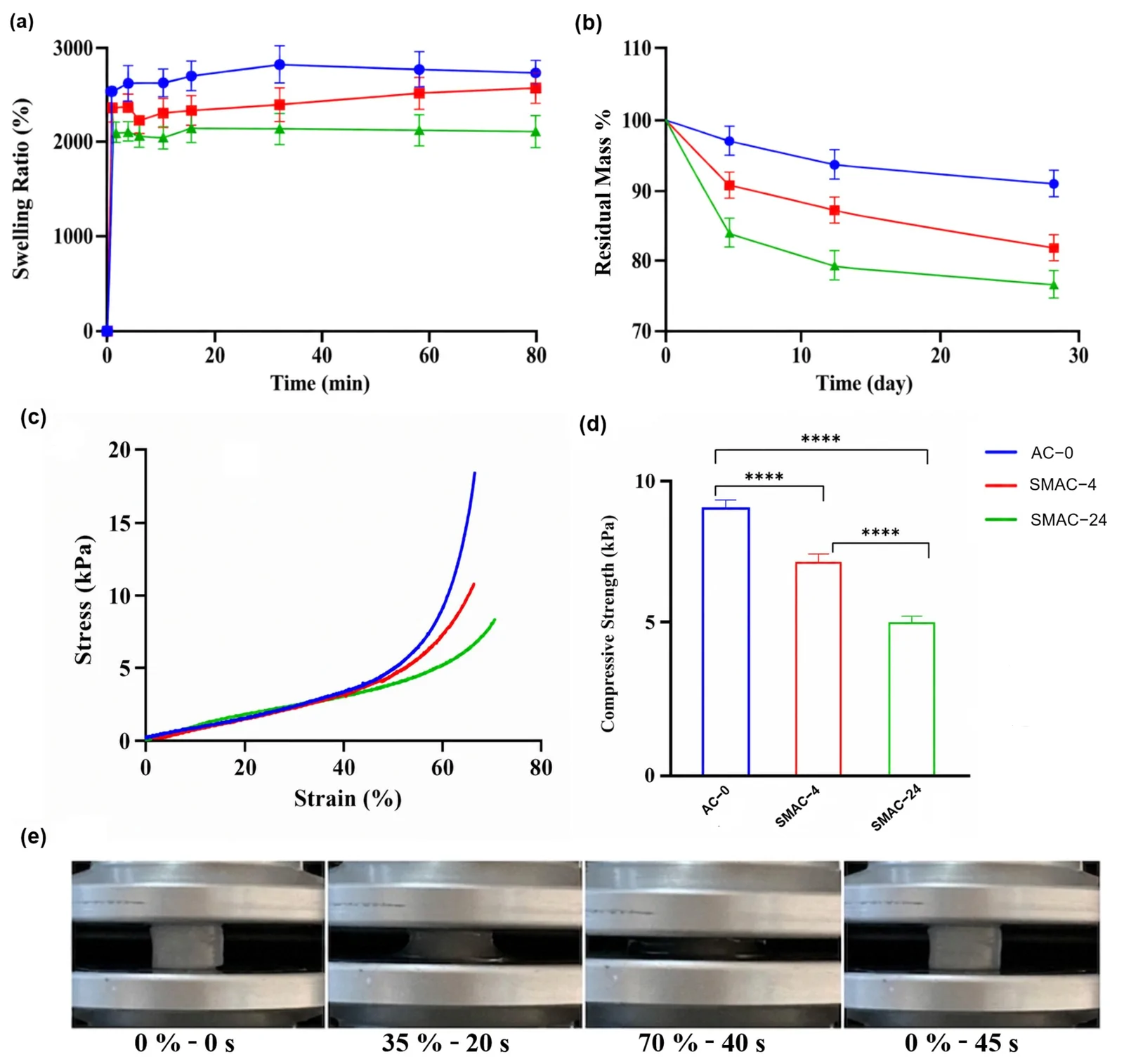
Characterization and performance data for cryogels: (**a**) time-dependent swelling degree (%) graph for pure and surface-modified agarose samples, (**b**) in vitro degradation (mass loss) profiles over 28 days, (**c**) stress–strain curves illustrating the mechanical strength of cryogel formulations, (**d**) compressive stress values of the samples at 60% strain, and (**e**) digital photographs showing the physical deformation of a typical cryogel sample during a compression test and the recovery process after the force is removed. Statistical significance is indicated by **** *p* < 0.0001.

As depicted in Figure 2a, all cryogels exhibited an ultra-rapid water uptake profile, reaching thermodynamic swelling equilibrium within the first 5 min of exposure to the physiological PBS medium (pH 7.4). This rapid swelling transition indicates the presence of supermacroporous cryogel networks, where fluid infiltration is driven by immediate capillary suction through open, interconnected pore channels, rather than the slow, diffusion-limited hydration typical of conventional hydrogels. Quantitatively, native AC-0 achieved a maximum swelling ratio of ~2750%. Upon surface modification, a systematic charge-dependent decrease in the fluid retention capacity was observed. SMAC-4 stabilized at ~2400%, whereas the highly charged SMAC-24 showed the lowest equilibrium capacity at ~2100%. This inverse relationship between the surface charge density and swelling capacity aligns with polymer network thermodynamics. The covalent immobilization of bulky, highly hydrophilic quaternary ammonium headgroups increases the localized mass density along pore struts. This chemical grafting reduces the net free volume available within the internal macropores and simultaneously rigidifies the local agarose wall segments, suppressing the elastic expansion capacity of the porous matrix and resulting in a controlled reduction in the total fluid capacity.

The long-term physiological durability of the scaffolds was monitored over a 28-day incubation period (Figure 2b). Native AC-0 exhibited good structural resilience, retaining over 92% of its initial mass by day 28, confirming the strong physical entanglement and hydrogen-bonded stability of the unfunctionalized marine polysaccharide frame. Following quaternization, the hydrolytic degradation rate exhibited systematic acceleration. The final remaining mass values decreased to approximately 82% for SMAC-4 and 76% for SMAC-24. This accelerated degradation curve directly stems from the disruption of native interchain hydrogen bonds. As the hydroxyl sites are progressively consumed and substituted by the highly positive quaternary ammonium moieties, the local crystalline double-helix bundles of agarose are partially forced apart by the electrostatic repulsion. This structural loosening facilitates deeper penetration of water molecules into the load-bearing struts, accelerating the hydrolytic cleavage of beta-1,4 and alpha-1,3-glycosidic linkages over extended durations. These outcomes clearly demonstrate that the degree of surface functionalization serves as a critical design parameter governing not only the chemical modification density but also the long-term degradation kinetics of post-quaternized agarose cryogels.

The mechanical resilience profiles obtained via uniaxial compression testing are presented in Figure 2c,d. Quantitatively, native AC-0 at 60% strain exhibited the highest ultimate compressive strength at 9.2 kPa. In complete validation of our room-temperature synthetic rationale, the quaternized derivatives successfully avoided the structural collapse observed at 50 °C. SMAC-4 and SMAC-24 maintained highly robust mechanical architectures, yielding compressive strengths of 7.4 kPa and 5.0 kPa at 60% strain, respectively (Figure 2d). The slight, controlled reduction in the compressive strength from AC-0 to SMAC-24 is a direct mechanical manifestation of the intra-strut electrostatic repulsions introduced by the dense accumulation of quaternary ammonium charges, which marginally lowers the buckling resistance of the individual pore walls under load. As shown in Figure 2e, the initial cylindrical specimens (0–0 s) exhibited homogeneous volumetric deformation after 35% compression (35–20 s) and maintained their structural integrity without any fracture or crack formation, even at a high compression rate of 70% (70–40). The rapid return of cryogels to their original cylindrical geometry and dimensions immediately (5 s) upon load release at room temperature was macroscopic evidence of shape-memory elastomeric compliance. This robust mechanical performance ensures that the optimized SMAC matrices can effectively withstand the physical stresses encountered in dynamic wound care and tissue engineering.

### 2.5. Microstructural Morphology and Elemental Mapping

**Figure 3:**
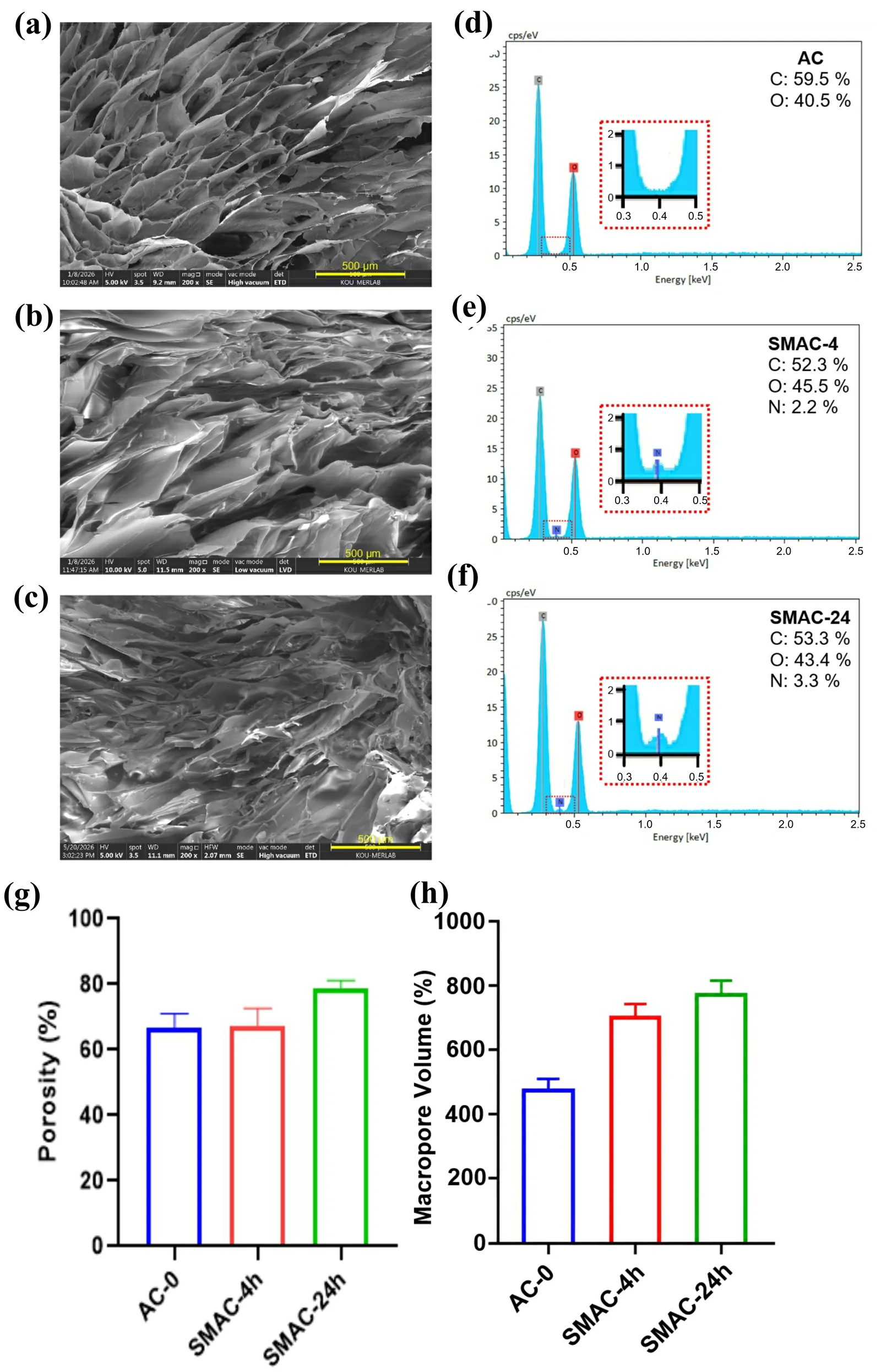
Characterization of pure agarose cryogel and surface-modified agarose cryogels (SMAC-4, SMAC-24): (**a**–**c**) SEM images showing the internal pore structure of the samples; (**d**–**f**) EDX spectra confirming the elemental composition; (**g**) average porosity %; and (**h**) macropore volume fractions.

The internal architectural alignment, pore connectivity, and localized elemental distributions of the cryogels were thoroughly investigated using SEM coupled with EDX. Figure 3 presents the cross-sectional SEM micrographs and corresponding EDX spectra for native AC-0 (Figure 3a), SMAC-4 (Figure 3b), and SMAC-24 (Figure 3c), along with the statistical quantification of the average pore diameters (Figure 3d) and cumulative macropore volume profiles (Figure 3e).

The SEM micrographs demonstrate that the post-synthetic room-temperature modification route preserves the delicate, interconnected supermacroporous networks generated by the ice-templating process. Native AC-0 (Figure 3a) exhibited highly organized, continuous, sheet-like polymeric walls outlining well-defined macropores. Upon heterogeneous surface quaternization for 4 h (SMAC-4, Figure 3b) and 24 h (SMAC-24, Figure 3c), the overall microstructural skeleton remained preserved, with no apparent signs of wall collapse or fragmentation. The porosity increased from 66.7% for AC-0 to 67.3% for SMAC-4 and 78.7% for SMAC-24 (Figure 3d), indicating that surface quaternization did not compromise the interconnected supermacroporous architecture. This increase in porosity, particularly for SMAC-24, may be attributed to electrostatic repulsion introduced by the cationic quaternary ammonium groups and enhanced water uptake associated with their hydrophilic nature, which may promote local expansion of the modified polymer network and contribute to the increased macropore volume fraction. The increased porosity observed after quaternization may be advantageous for wound-care applications by providing greater macropore volume for fluid uptake and retention, while maintaining an open scaffold structure favorable for tissue-material interaction. Nevertheless, the direct contribution of these structural features to exudate management and wound healing will require further evaluation.

To directly confirm that the tracked nitrogen accumulation originated from the covalent immobilization of CHPTAC segments rather than physical entrapment, point-mode EDX analysis was conducted across the solid pore walls. The EDX spectrum of native AC-0 (Figure 3a) displays only two prominent signals corresponding to carbon (C) and oxygen (O), representing the initial carbohydrate frame of agarose. Following alkaline activation and etherification, the EDX spectra of SMAC-4 (Figure 3b) and SMAC-24 (Figure 3c) clearly show the emergence of a distinct nitrogen (N) peak located at approximately 0.39 keV. The absolute intensity and net counts of the nitrogen signal increased from 4 h to 24 h, providing direct qualitative and microanalytical validation for the successful execution of the post-synthetic quaternization framework. This spatial elemental distribution confirms that the active cationic sites are uniformly bound along the exposure surfaces of the pore walls, establishing an ideal structural layout for contact-active biological interactions.

### 2.6. In Vitro Antibacterial and Growth Inhibition Efficacy

**Figure 4:**
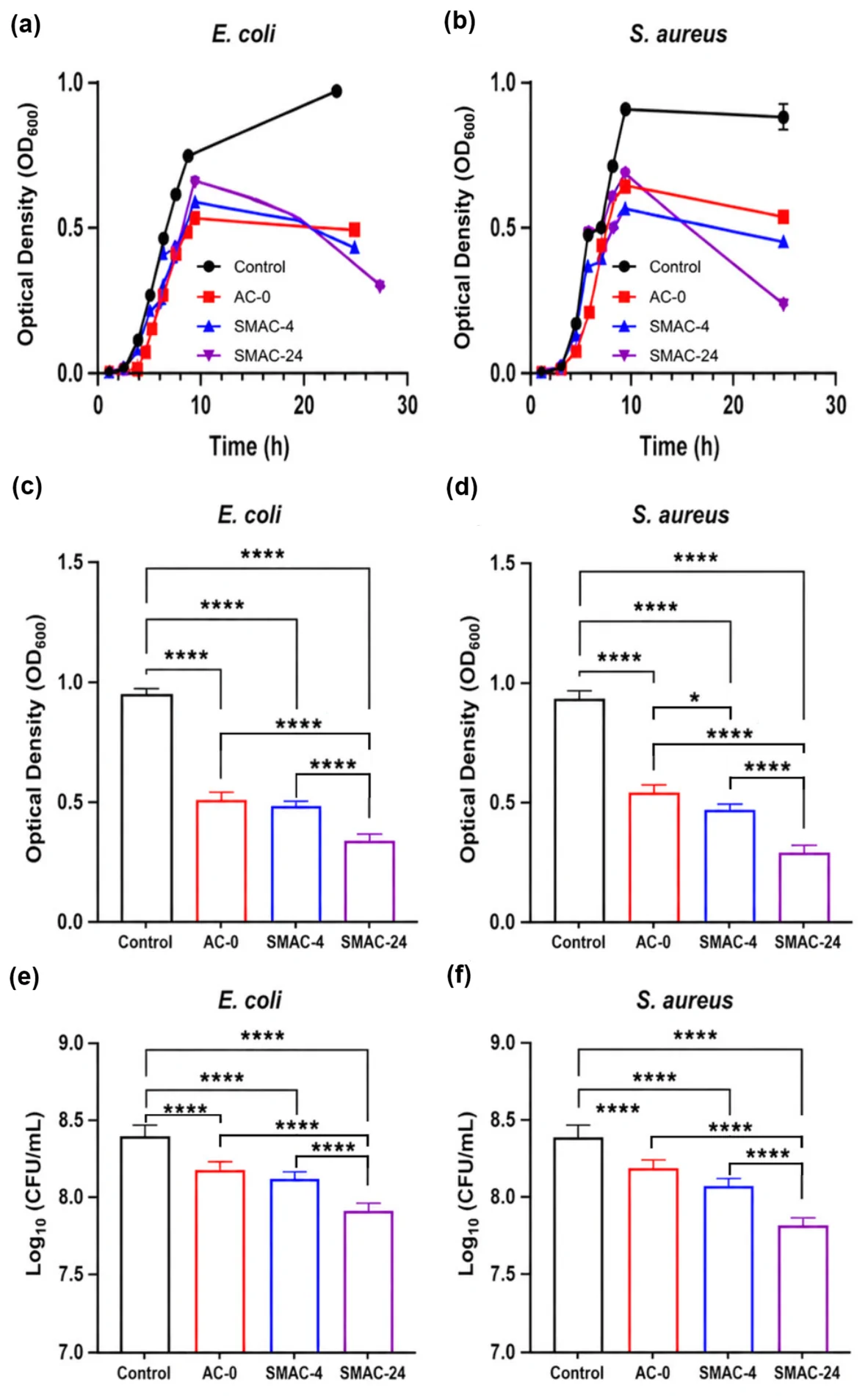
Antibacterial activity of AC-0, SMAC-4 and SMAC-24 on *E. coli* and *S. aureus*. (**a**,**b**) Time-course of bacterial growth monitored via OD_600_; (**c**,**d**) OD_600_ values measured at 24 h; and (**e**,**f**) viable bacterial counts expressed as Log_10_(CFU/mL) at 24 h. Values are expressed as mean ± SD of three independent experiments (*n* = 3). Statistical significance was determined relative to the untreated bacterial control and is indicated by * *p* < 0.1 and **** *p* < 0.0001.

The antibacterial activity of synthesized agarose cryogel (AC-0) and its surface quaternized derivatives (SMAC-4 and SMAC-24) against Gram-negative *E. coli* and Gram-positive *S. aureus* was evaluated using time-dependent growth curves and colony-forming unit (CFU) analyses. The findings indicate that surface quaternization is a key functional modification that significantly suppressed bacterial proliferation. When the time-dependent growth curves were examined (Figure 4a,b), it was observed that the control groups exhibited a typical logarithmic growth profile for both bacterial species. In contrast, a significant inhibition of bacterial growth was observed in the presence of cryogel samples. Specifically, the SMAC-24 sample showed the highest antibacterial effect, exhibiting the lowest optical density values during 24 h of incubation (Figure 4c,d). When interpreted according to the classical bacterial growth model, the time-dependent OD_600_ curves indicate that the inhibitory effect of the cryogel derivatives was not primarily due to complete suppression of the initial adaptation phase. In the early incubation period, both bacterial strains still showed an increase in OD_600_, suggesting that initial metabolic adaptation and early proliferation were not fully prevented. However, the divergence between the untreated control and the cryogel-treated groups became more evident during the later stages of the logarithmic growth phase and the transition to the stationary phase. This effect was most pronounced for SMAC-24, which showed the lowest OD_600_ values at 24 h for both *E. coli* and *S. aureus*. The corresponding reduction in Log_10_(CFU/mL) values further supports that the maximum inhibitory effect of SMAC-24 occurred during the late logarithmic/stationary-transition stage, when cumulative bacterial biomass formation and viable cell recovery were markedly reduced. The partial OD reduction observed in the AC-0 sample is thought to be due to the capacity of the cryogel’s supermacroporous structure to physically trap bacteria, rather than an antibacterial effect. This suggests that pure agarose does not exhibit intrinsic antibacterial activity but may create a physical structure-based restriction effect [30,31].

The significant increase in inhibition observed with increasing quaternization time is directly related to the density of quaternary ammonium groups on the surface. CFU analyses (Figure 4e,f) confirm that SMAC-24 cryogel statistically significantly reduces bacterial colonies (*p* < 0.001). The strong reduction in CFUs observed for the surface-quaternized cryogels is consistent with a contact-active antibacterial mechanism previously reported for immobilized quaternary ammonium surfaces. The positively charged quaternary ammonium groups are expected to interact electrostatically with the negatively charged components of the bacterial envelope, which may compromise the integrity of the membrane [31,32].

**Figure 5:**
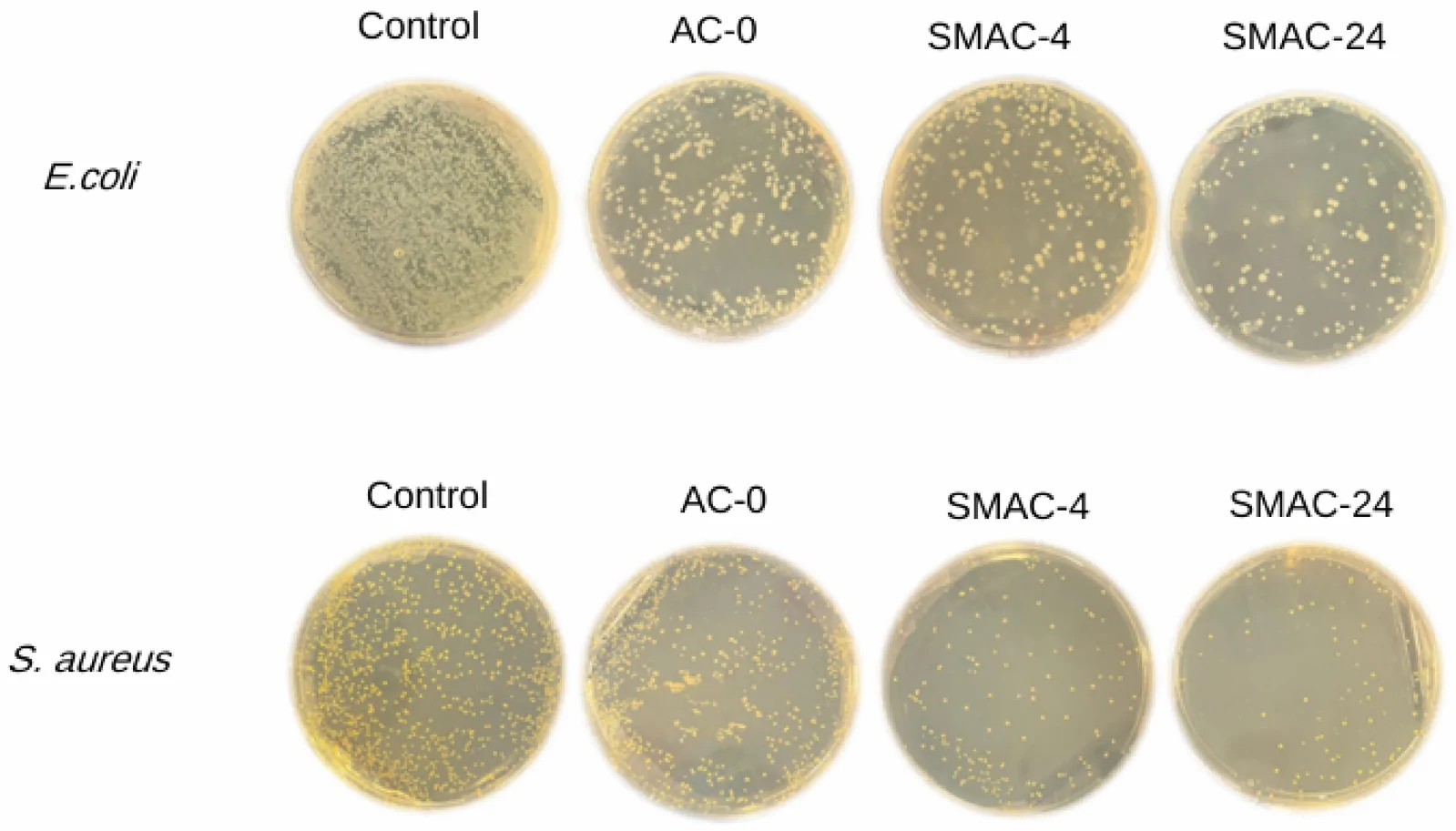
Images of colony formation of *E. coli* and *S. aureus* on agar plates after treatment with different sample groups (Control, AC-0, SMAC-4, and SMAC-24).

Plate images (Figure 5) visually support these results. While dense colony formation was observed in the control groups, only a partial decrease in colony count was detected in AC-0 samples. This result confirms that pure agarose does not possess antibacterial properties and can only show a limited physical retention effect due to its porous structure. In contrast, colony density was significantly reduced in samples subjected to surface quaternization (SMAC-4 and SMAC-24), with almost complete inhibition achieved for both bacterial species, especially in the SMAC-24 sample. This superior performance of SMAC-24 indicates that the contact-activated killing mechanism reaches its maximum efficacy when the quaternary ammonium group density on the surface reaches a critical threshold value [31,33]. Overall, when Figure 4 and Figure 5 are considered together, it is seen that the antibacterial performance of quaternized agarose cryogels is determined by two main components: (i) the positive charge density on the surface and (ii) the macroporous structure increasing the contact area with bacteria. The observation of similarly strong efficacy against both *E. coli* and *S. aureus*, particularly in the SMAC-24 sample, demonstrates that the developed system has broad-spectrum antibacterial potential. This supports the idea that the material can create a suitable microenvironment for tissue regeneration by providing infection control in chronic wound settings.

### 2.7. In Vitro Cytotoxicity

**Figure 6:**
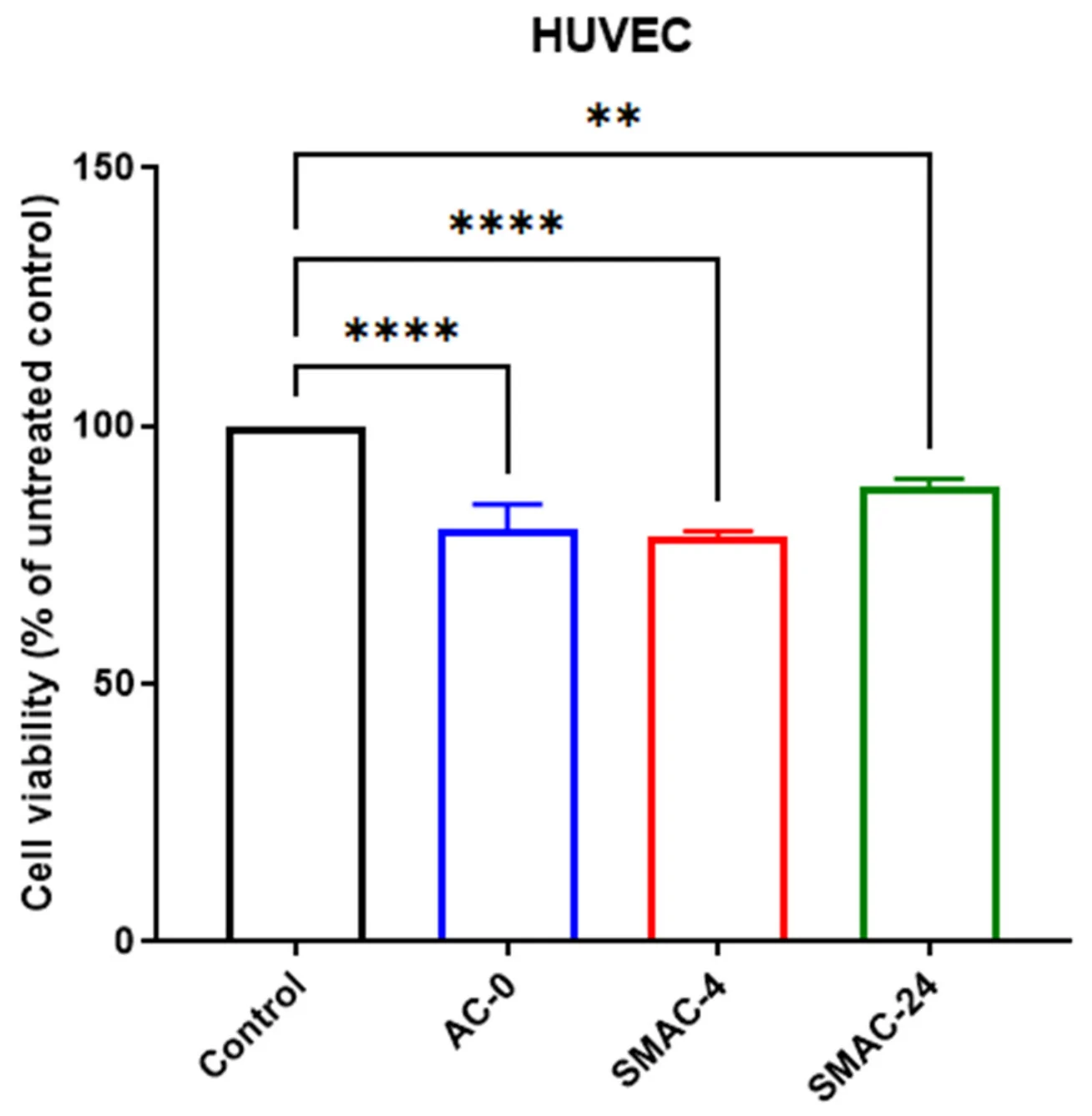
In vitro cytocompatibility of AC-0, SMAC-4 and SMAC-24 cryogels by means of MTT assay. The viability of HUVEC after 24 h of exposure to the cryogel extracts was determined and expressed as a percentage relative to the untreated control. Data are expressed as the mean ± SD of three independent experiments (*n* = 3). Statistical significance versus untreated control was assessed using one-way ANOVA followed by Dunnett’s multiple-comparison test. ** *p* < 0.01 and **** *p* < 0.0001.

After the physicochemical and antibacterial characterization of the surface-quaternized cryogels, their cytocompatibility was tested with HUVECs to evaluate their possible suitability for biomedical applications. The indirect cytotoxicity of AC-0, SMAC-4 and SMAC-24 was determined by means of an MTT assay after 24 h exposure to cryogel extracts, and the cell viability was expressed in relation to the untreated control (Figure 6). None of the tested cryogel extracts reduced the HUVEC viability below the threshold of 70% used for in vitro cytotoxicity assessment. The cell viability of unmodified agarose cryogel (AC-0) was 80.0 ± 4.7% and that of the SMAC-4 extract was similar at 78.5 ± 1.0%. Interestingly, SMAC-24 showed the highest cell viability among the tested cryogel formulations, with 88.1 ± 1.6% viability after 24 h of exposure. Thus, extended surface quaternization under the experimental conditions of the present study did not result in increased extract-mediated cytotoxicity.

Cytocompatibility of cationically modified biomaterials is an important consideration because the biological response to polycationic materials can vary based on the polymer structure and the nature and density of cationic groups [18]. Therefore, the increase in antibacterial activity by quaternization must be balanced against possible negative effects on mammalian cells. Previous studies on quaternized polysaccharide-based materials have shown that the appropriate cationic modifications can also endow antibacterial activity while maintaining favorable biological performance [34,35,36]. In particular, the biological effects of polysaccharide quaternization depend strongly on the chemical characteristics and the extent of the modification, which emphasizes the importance of evaluating the cytocompatibility for each individual material system [36]. This balance was particularly evident for SMAC-24 in the present study. SMAC-24 showed the strongest antibacterial activity against both *E. coli* and *S. aureus* but also exhibited the highest HUVEC viability among the cryogel formulations investigated. This is an important observation, because it shows that the increased antibacterial effect obtained by prolonged surface quaternization did not come with a corresponding increase in cytotoxicity. Thus, the combination of strong antibacterial activity and favorable HUVEC viability identifies SMAC-24 as the most promising formulation among the tested cryogels from the biological performance point of view.

The observed positive cytocompatibility of the surface-quaternized cryogels might also be linked to the post-synthetic functionalization strategy used in this study, where quaternary ammonium groups were covalently attached to the preformed agarose matrix instead of being incorporated as freely diffusible antibacterial agents. However, the current MTT assay is an evaluation of the cell response to the components extractable from the cryogels and does not directly evaluate cell–material interactions. Thus, although the results indicate good short-term extract cytocompatibility, future studies using direct-contact assays, longer exposure times, and additional cellular end-points are needed to fully establish the biocompatibility of these materials for wound-contact and tissue engineering applications.

## 3. Conclusions

In this study, prefabricated agarose cryogels were successfully functionalized to generate surface-modified agarose cryogels (SMACs) with contact-active antibacterial functionality through post-synthetic quaternization with CHPTAC. The surface modification enabled covalent immobilization of quaternary ammonium groups while preserving the interconnected supermacroporous architecture of the cryogels. The degree of surface quaternization emerged as an important design parameter, influencing antibacterial activity as well as the swelling and mechanical properties of the scaffolds. Among the tested formulations, SMAC-24 exhibited the strongest antibacterial activity against both *Escherichia coli* and *Staphylococcus aureus*, together with favorable short-term HUVEC cytocompatibility, with 88.1 ± 1.6% viability after 24 h of exposure to scaffold extracts. These findings demonstrate that post-synthetic surface quaternization provides a practical strategy for converting bioinert agarose cryogels into non-leaching antibacterial scaffolds while retaining their key porous structural features.

However, the negative effects of prolonged surface quaternization on swelling behavior and mechanical performance were considered as aspects requiring optimization of the system. In particular, while the present study establishes the chemical, structural, and in vitro antibacterial performance of SMAC scaffolds, a validation of biocompatibility and wound healing performance in appropriate in vivo models remains the critical next milestone. In future studies, systematically optimizing the reaction parameters, investigating the effects of different degrees of quaternization on biological performance, conducting long-term in vitro stability studies, and validating biocompatibility and wound healing performance in appropriate in vivo models will be important for revealing the clinical translation potential of the developed biomaterial.

## 4. Materials and Methods

### 4.1. Materials

Agarose (low EEO, bioreagent grade) and phosphate-buffered saline (PBS, pH 7.4) tablets were purchased from Sigma-Aldrich (St. Louis, MO, USA). The cationic precursor, (3-chloro-2-hydroxypropyl)trimethylammonium chloride (CHPTAC, 65 wt% aqueous solution), was obtained from Tokyo Chemical Industry (TCI, Tokyo, Japan). Sodium hydroxide (NaOH) pellets were purchased from Merck (Darmstadt, Germany), while hydrochloric acid (HCl, 37%, analytical grade) was supplied by Tekkim (Türkiye). For the antimicrobial assays, *Escherichia coli* (ATCC 25922) and *Staphylococcus aureus* (ATCC 25923) strains were obtained from the American Type Culture Collection (ATCC, Manassas, VA, USA). All cell culture media, nutrient broths, and related biological reagents were purchased from Thermo Fisher Scientific (Waltham, MA, USA). Ultra-pure water was utilized throughout all experimental procedures. Unless otherwise stated, all chemical reagents were used as received without further purification.

### 4.2. Synthesis and Post-Functionalization of Agarose Cryogels

Pure agarose cryogels were fabricated by adapting a cryotropic gelation protocol established in the literature [22]. Briefly, a specific amount of agarose powder (0.03 g/mL) was dissolved in distilled water at 90 °C under magnetic stirring until a homogeneous solution was obtained and gradually cooled to 55 °C to prevent premature gelation. Then, the solution pH was adjusted to around 12.0 using 1 N NaOH at 55 °C. Subsequently, 1.5 mL aliquots of the warm agarose solution were transferred into syringe molds and frozen (−21 °C, 1 h), followed by incubation (−5 °C, overnight) to promote cryogel formation. The cryogels were then thawed at room temperature and sequentially washed with graded ethanol solutions (50%, 75%, and 100%) to remove residual impurities, before extensive rinsing with distilled water until a neutral pH was achieved. In the final step, the samples were frozen at −80 °C and lyophilized.

To synthesize the cationic derivatives, the pre-fabricated, dried pure agarose cryogels were functionalized via a post-synthetic heterogeneous etherification strategy. The dry cryogel scaffolds were initially dispersed and allowed to re-swell completely in ultra-pure water inside a reaction flask under gentle magnetic stirring at a designated temperature. Subsequently, a predetermined volume of aqueous NaOH solution was introduced dropwise to catalyze the deprotonation of the agarose hydroxyl groups and promote the in situ conversion of the cationic precursor into its active epoxide intermediate (epoxypropyltrimethylammonium chloride, EPTAC). Following alkaline activation, specified amounts of the CHPTAC solution were added slowly and carefully to the reaction mixture using a syringe, in accordance with the experimental design detailed in Table 1.

The heterogeneous reaction was carried out for 4 h (SMAC-4), 6 h (SMAC-6), and 24 h (SMAC-24) under controlled thermal and kinetic conditions. Upon completion of the targeted reaction period, the quaternized cryogels were isolated from the reaction flask and immediately immersed in a large volume of deionized water to quench the etherification. The modified scaffolds were subjected to exhaustive rinsing with distilled water until a constant neutral pH was achieved. The final quaternized cryogels were then re-lyophilized for structural and biological evaluations.

### 4.3. Chemical and Elemental Characterization

#### 4.3.1. Fourier Transform Infrared (FTIR) Spectroscopy

The chemical surface functionalities and the verification of the covalent grafting process of dry cryogels were monitored using a Bruker Alpha-P FTIR spectrophotometer (Bruker Optics GmbH, Ettlingen, Germany) equipped with an Attenuated Total Reflection (ATR) module.

#### 4.3.2. Elemental Analysis and Degree of Substitution (DS_EA_)

To quantitatively determine the mass fractions of nitrogen (N) within the dry cryogels, high-precision combustion elemental analysis was performed using an EA-IRMMS system (Thermo Finnigan MAT 253). The degree of substitution (DS_EA_), defining the average number of cationic groups attached per repeating disaccharide unit of the agarose chain, was calculated based on the experimentally derived nitrogen content (N%) according to Equation (1):

(1) $${DS}_{EA}=\frac{153\times \%N}{1400-(152\times \%N)}$$

where 153 g/mol represents the average molecular weight of the unmodified agarose repeating unit (anhydrodisaccharide unit), %N is the statistically determined mass percentage of nitrogen on a strictly dry weight basis, 152 accounts for the net molecular weight increment introduced by the covalently attached 2-hydroxypropyltrimethylammonium chloride substituent after subtracting the leaving group, and 1400 is a constant conversion factor derived from the atomic weight of nitrogen (14 g/mol × 100).

### 4.4. Swelling and Degradation Properties

The water-intake capacity and swelling kinetics of the cryogels were quantified at room temperature via a conventional gravimetric method in phosphate-buffered saline (PBS, 0.1 M, pH 7.4) to simulate physiological fluid conditions. The initial weights of the completely dry, lyophilized cylindrical cryogel monoliths were accurately recorded as ($W_{i}$) using a high-precision analytical balance. The scaffolds were then completely submerged in the PBS medium to allow capillary-driven fluid absorption. At predetermined time intervals until the swelling reached strict thermodynamic equilibrium, the specimens were carefully removed from the buffer solution. The excess, non-bound surface water was gently blotted away using a damp filter paper to avoid withdrawing water from the internal macropores. The fully swollen weight ($W_{s}$) of each sample was then recorded. The equilibrium swelling ratio (%) was calculated according to Equation (2):

(2) $$Swelling\;Ratio\;\left(\%\right)=\frac{W_{s}-W_{i}}{W_{i}}\;\times \;100$$

To evaluate the structural stability and mass loss behavior of the cryogels under simulated physiological conditions, in vitro hydrolytic degradation assays were conducted over an extended period of 4 weeks. Lyophilized cryogel discs of known initial dry weight ($W_{d}$) were placed into individual 10 mL centrifuge tubes containing a fixed volume of PBS solution (0.1 M, pH 7.4). The sealed tubes were maintained in an incubator at 37 °C. At designated temporal checkpoints (1, 2, 4 weeks), the respective cryogel matrices were removed from the incubation medium. To stop any ongoing degradation and eliminate residual buffer salts, the isolated monoliths were thoroughly rinsed with ultrapure water. The samples were then re-frozen at −80 °C and completely re-lyophilized. The final dry weight ($W_{f}$) at each time point was recorded, and the cumulative degradation ratio (%) was determined according to Equation (3):

(3) $$Degradation\;Ratio\;\left(\%\right)=\frac{W_{d}-W_{f}}{W_{d}}\;\times \;100$$

### 4.5. Morphological and Mechanical Characterization

The morphological examinations of the cryogels were performed using a scanning electron microscope (SEM; Bruker Nano GmbH, Berlin, Germany). Before analysis, the lyophilized cryogel samples were cut into appropriate sizes and coated with a thin layer of gold to ensure electrical conductivity. Micrographs were acquired under high vacuum at an acceleration voltage of 20 kV to evaluate porosity. Concurrently performed EDX analysis mapped the elemental distribution within the modified cryogels.

The mechanical strength properties of the cryogels were determined using uniaxial free compression tests conducted at room temperature using an MTS 810 Material Testing System. The cylindrical cryogel specimens used in the tests had a diameter of 11 mm and a height of 5 mm. Before testing, the dry cryogel matrices were fully re-swollen in PBS until equilibrium was reached. Each specimen was compressed at a constant rate of 1 mm/min using a 10 kN load cell until a 70% strain relative to the initial height was reached. To ensure reproducibility, three parallel measurements were performed for each formulation. The Young’s modulus (E), a measure of the cryogels’ elasticity, was calculated from the slope of the initial linear region of the stress–strain curve.

### 4.6. Antibacterial Activity

The antibacterial activity of the synthesized surface-quaternized agarose cryogels was evaluated against Gram-negative *Escherichia coli* and Gram-positive *Staphylococcus aureus*. Cryogel samples (8 mm diameter by 2 mm thickness) were prepared as disks and sterilized using UV radiation. The antibacterial performance was assessed using the dynamic contact method (modified ASTM E2149-13a) combined with a colony-counting assay. Bacterial cultures were incubated in Müller–Hinton broth at 37 °C for 24 h and adjusted to approximately 10^8^ CFU/mL (OD_600_ = 0.1–0.2). Aliquots (10–50 µL) of bacterial suspension were applied directly onto the surface of dry cryogel samples and incubated at 37 °C for 30–120 min to allow bacteria–material contact. Bacterial growth kinetics were monitored by measuring OD_600_ at predetermined time intervals.

Following incubation, the cryogel samples were transferred to sterile PBS for recovery of adherent bacteria, followed by serial dilution and plating onto Müller–Hinton agar. After incubation at 37 °C for 24 h, viable colonies were counted and antibacterial (bactericidal) activity of the cryogels based on CFU reduction was calculated using Equation (4):

(4) $$Antibacterial\;Effect\;\left(\%\right)=\frac{C_{0}-C}{C_{0}}\;\times \;100$$

where *C*_0_ represents the number of bacterial colonies in the control group (unmodified agarose cryogels), while *C* represents the number of colonies recovered after exposure to the modified cryogels. All experiments were performed in triplicate.

### 4.7. In Vitro Cytotoxicity Analysis

For the in vitro cytotoxicity assessment of the surface-quaternized agarose cryogels, biomaterial extracts were prepared under serum-free conditions to assess the potential effects of the extractable components on the viability of human umbilical vein endothelial cells (HUVEC) by following the previously described protocol [7]. In brief, the cryogel samples were incubated in FBS-free cell culture medium at a solid material-extraction medium ratio of 10 mg mL^−1^ for 24 h at 37 °C in a humidified atmosphere with 5% CO_2_. After extraction, the media were collected and used immediately for cytotoxicity assays. HUVECs (PCS-100-010) were seeded in 96-well plates at a density of 2 × 10^4^ cells/well and incubated at 37 °C in a 5% CO_2_ humidified atmosphere for 24 h to allow cell attachment. The culture medium was aspirated, and 100 µL of the respective cryogel extract was added. Untreated control cells were kept in the same culture medium without exposure to cryogel extracts and considered to represent 100% viability. Cells were then treated with the extracts for an additional 24 h under the same culture conditions.

Cell viability was determined using an MTT assay after exposure. Briefly, 10 μL of MTT reagent was added to each well and incubated at 37 °C in 5% CO_2_ for the formation of formazan crystals. Then, the medium containing MTT was aspirated, and 100 µL of dimethyl sulfoxide (DMSO) was added to each well to dissolve the resulting formazan crystals. The solution was mixed gently and allowed to solubilize completely. The absorbance was read at 570 nm using a microplate reader. Cell viability was assessed relative to the untreated control using the following equation:

(5) $$Cell\;viability\;\left(\%\right)=\frac{A_{570},sample}{A_{570},control}\;\times \;100$$

where A_570_, sample is the absorbance of cells treated with the cryogel extract and A_570_, control is the absorbance of untreated control cells. Each experimental condition was analyzed in three independent experiments (n = 3) and data are shown as mean ± standard deviation (SD).

### 4.8. Statistical Analysis

All quantitative experiments were conducted in three independent replicates (n = 3) and data are presented as mean ± standard deviation (SD). Statistical analyses were conducted using GraphPad Prism version 9.3 (GraphPad Software, San Diego, CA, USA). Time-dependent bacterial growth data (OD_600_) were analyzed separately for each bacterial species using two-way analysis of variance (ANOVA) with treatment and incubation time as the two factors, followed by Tukey’s multiple-comparison test to compare each cryogel-treated group with the corresponding control. One-way ANOVA followed by Tukey’s multiple-comparison test was used to analyze endpoint OD_600_ values and viable bacterial counts [Log _10_ (CFU/mL)] for each bacterial species separately. HUVEC cell viability MTT assay data were analyzed by means of one-way ANOVA with Tukey’s multiple-comparison test comparing each cryogel extract-treated group to the untreated cell control. Differences were regarded as being significant at *p* < 0.05. Statistical significance is indicated in the figures as * *p* < 0.05, ** *p* < 0.01, *** *p* < 0.001 and **** *p* < 0.0001.

## Figures and Tables

**Table 1:** Synthesis parameters and elemental composition of surface-modified agarose cryogels (SMAC).

Samples	Agarose Cryogels Conc. (mg/mL)	Time (h)	Temperature (°C)	CHPTAC/Agarose Ratio (mol/g)	NaOH/ CHPTAC Ratio (mol/mol)	N (%)	DS_EA_
AC-0	4	0	-	-	-	0.00	0.00
SMAC-4	4	4	RT	10.39	2.09	0.41	0.05
SMAC-6	4	6	RT	10.39	2.09	0.49	0.06
SMAC-24	4	24	RT	10.39	2.09	0.90	0.11

CHPTAC, 3-chloro-2-hydroxypropyltrimethylammonium chloride; NaOH, sodium hydroxide.

## Data Availability

The data presented in this study are available upon request from the corresponding authors.

## Supplementary Materials

The following supporting information can be downloaded at https://www.mdpi.com/article/10.3390/gels12090792/s1. Table S1: Optimization matrix and elemental composition of post-modified agarose cryogels at constant temperature (50 °C).
