# Supplementary material for: Post-Synthetic Surface Quaternization of Agarose Cryogels: Optimizing Structural Integrity and Broad-Spectrum Growth Inhibition
Source: Gels. 2026 Sep 1;12(9):792. doi: 10.3390/gels12090792 (PMC13606618; doi:10.3390/gels12090792)
Supplement: Supplementary file 1 [file gels-12-00792-s001.zip › gels-4457154-supplementary.pdf]

Supplementary Information

Post-Synthetic Surface Quaternization of Agarose Cryogels: Optimizing Structural Integrity and Broad-Spectrum Growth Inhibition

Ahmet Erdem* [1], Elif Beyza Eren [1, 2], Farouk Segujja [1], Elif Kale Bakir [3], Yonca Yüzügüllü Karakus [3], Süheda Ercek [2], Tugba Dispinar Gezer* [2]

1 Department of Biomedical Engineering, Faculty of Technology, Kocaeli University, 41001 Kocaeli, Turkey

2 TUBITAK UME, TUBITAK, Gebze Yerleşkesi, 41470 Gebze, Kocaeli, Turkey

3 Department of Biology, Faculty of Science and Arts, Kocaeli University, 41001 Kocaeli, Turkey

Table S1: Optimization matrix and elemental composition of post-modified agarose cryogels at constant temperature (50 °C)

ID	Time (h)	Agarose cryogel (mg/mL)	CHPTAC/Agarose cryogel ratio (mol/g)	NaOH:CHPTAC ratio (mol/mol)	N (%)	DS_{EA}
SMAC-1a50	1	1	0.135	2	0.64	0.08
SMAC-1b50	1	2	0.067	2	0.68	0.08
SMAC-1c50	1	4	0.034	2	0.70	0.08
SMAC-2a50	2	1	0.135	2	0.8	0.10
SMAC-2b50	2	2	0.067	2	0.83	0.10
SMAC-2c50	2	4	0.034	2	0.94	0.11
SMAC-4c50	4	4	0.034	2	1.09	0.14
SMAC-6c50	6	4	0.034	2	1.18	0.15
